# The G protein coupled receptor CXCR4 designed by the QTY code becomes more hydrophilic and retains cell signaling activity

**DOI:** 10.1038/s41598-020-77659-x

**Published:** 2020-12-07

**Authors:** Lotta Tegler, Karolina Corin, Horst Pick, Jennifer Brookes, Michael Skuhersky, Horst Vogel, Shuguang Zhang

**Affiliations:** 1 Center for Bits and Atoms, Massachusetts Institute of Technology, Cambridge, MA 02139-4307 USA; 2 Molecular Biotechnology/IFM, Linköping University, 58183 Linköping, Sweden; 3 Biomedical Engineering Research Group, School of Electrical and Information Engineering, and Department of Molecular Medicine and Haematology, University of the Witwatersrand, Johannesburg, South Africa; 4 Institut des Sciences et Ingénierie Chimiques, Ecole Polytechnique Fédérale de Lausanne (EPFL), 1015 Lausanne, Switzerland; 5 Synthetic Neurobiology Group, Media Lab, Massachusetts Institute of Technology, Cambridge, MA 02139-4307 USA; 6 Present Address: Department of Chemistry and Biochemistry, University of California, Los Angeles, CA 90095-1570 USA; 7 Present Address: London Centre for Nanotechnology, University College London, 17-19 Gordon Street, London, WC1H 0AH UK; 8 Present Address: Biophysics, Computational Physics, Quantum Physics, University College London, London, UK

**Keywords:** Proteins, G protein-coupled receptors, Membrane proteins

## Abstract

G protein-coupled receptors (GPCRs) are vital for diverse biological functions, including vision, smell, and aging. They are involved in a wide range of diseases, and are among the most important targets of medicinal drugs. Tools that facilitate GPCR studies or GPCR-based technologies or therapies are thus critical to develop. Here we report using our QTY (glutamine, threonine, tyrosine) code to systematically replace 29 membrane-facing leucine, isoleucine, valine, and phenylalanine residues in the transmembrane α-helices of the GPCR CXCR4. This variant, CXCR4^QTY29^, became more hydrophilic, while retaining the ability to bind its ligand CXCL12. When transfected into HEK293 cells, it inserted into the cell membrane, and initiated cellular signaling. This QTY code has the potential to improve GPCR and membrane protein studies by making it possible to design functional hydrophilic receptors. This tool can be applied to diverse α-helical membrane proteins, and may aid in the development of other applications, including clinical therapies.

## Introduction

G protein-coupled receptors (GPCRs) are arguably the most important class of proteins. They are the largest class of receptors, and are involved in numerous physiological functions including sight, smell, inflammation, taste, and the immune response^1–5^. Moreover, they are involved in high threat and prolific diseases like cancer, HIV infection, and diabetes, and are the targets of ~ 50% of pharmaceutical drugs^3,6–11^.

The diverse and important functions of membrane proteins make them attractive templates for technological innovations. For example, photosystem 1 (PS1) can be integrated into chips to create photovoltaic cells^12^, and it has been hypothesized that olfactory receptors may be integrated into microfluidic devices to create smell sensors capable of detecting early-stage diseases and dangerous chemicals^13,14^. It may even be possible to design proteins de novo for novel applications.

One problem currently hampering the development of membrane protein-based, and particularly GPCR-based, technologies is the need to stabilize them with detergents outside of the cell membrane. Although various surfactants have traditionally been used, they often do not adequately stabilize the proteins for sufficient periods of time. Moreover, the optimal detergent for a given receptor must be empirically determined, requiring a significant amount of time and resources. An alternate approach would be to design GPCRs to become hydrophilic, thereby eliminating the need for detergents. Not only would this aid in the development of GPCR and membrane protein-based technologies, but it would also further build on the basic principles for de novo protein design.

With recent advancements in computational tools and software, researchers have started to design various proteins^15–17^. Several attempts with membrane proteins have focused on introducing hydrophilic amino acids like lysine or arginine to make them water-soluble. This goal has been accomplished with two small membrane proteins and one GPCR^18–22^. Although a computational approach was typically used^18–20^, one group substituted charged residues for hydrophobic ones^21^, and a second group “grafted” the hydrophilic face of a soluble protein onto the hydrophobic membrane-facing section of a membrane protein^22^. These strategies either require significant computational resources to design a water-soluble receptor, or may have limitations when applied to other membrane proteins. Moreover, detergents may still be needed for purification^18^.

**Figure 1:**
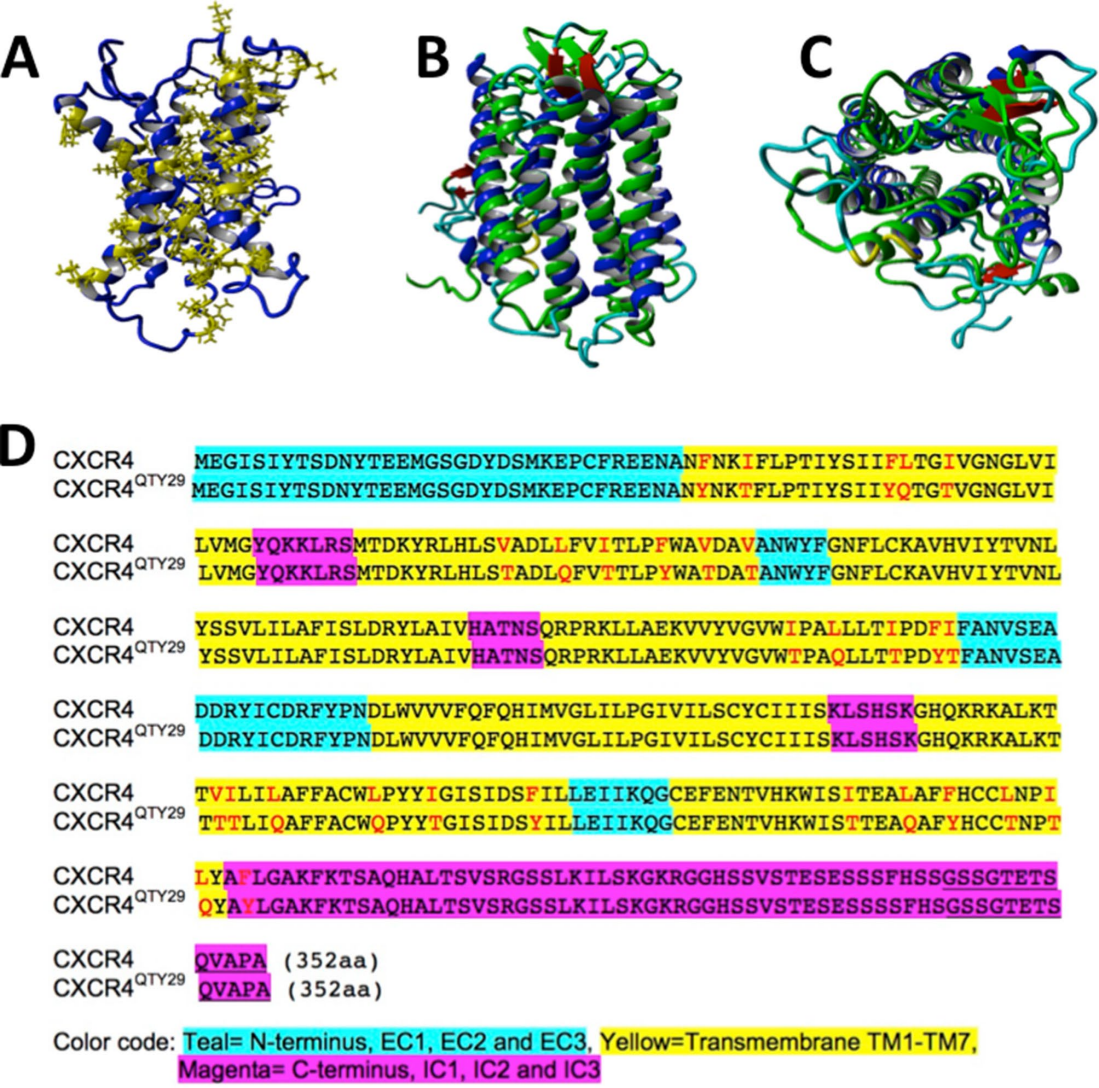
Schematic representations of CXCR4 and the proposed CXCR4^QTY29^ mutations. (**A**) Computational model of CXCR4^QTY29^. Hydrophobic residues are shown in yellow. The lipid-facing side faces the reader and the dimerization interface is on the other side. (**B**) Side view of CXCR4 (green) superimposed on the computational model of CXCR4^QTY29^ (blue). The helices nearly overlap, suggesting that the proposed mutations do not significantly alter the structure of CXCR4. (**C**) Extracellular view of the image in (**B**). The similarity in structures suggests that receptor binding and signaling may not be significantly affected. (**D**) Full-length sequence of the native CXCR4 aligned with CXCR4^QTY29^. The changed residues, which comprise only ~ 8% of the protein, are highlighted in red. A C-terminal GSSG linker and rho1D4 purification tag are underlined. The models in (**A**)–(**C**) were visualized within the simulation software YASARA^38^.

We previously reported an alternative approach for designing hydrophilic GPCRs, which we call the “QTY code”^23^. Using this code, hydrophobic amino acids are exchanged for hydrophilic residues with similar shapes and alpha-helix forming tendencies. Specifically, leucine (L) is replaced with glutamine (Q), valine (V) or isoleucine (I) are replaced with threonine (T), and phenylalanine (F) is replaced with tyrosine (Y) (Fig. 1). We showed that this code could be used to design hydrophilic chemokine receptors^23^. However, in order to make these receptors completely water-soluble, all hydrophobic transmembrane residues were replaced with non-charged hydrophilic ones. Such large-scale changes might not be tolerated as well by other GPCRs or helical membrane proteins. Moreover, the activity of these receptors in living cells was not determined.

Here we show that the QTY code can successfully be used to design a more hydrophilic form of CXCR4 that retains cellular signaling activity in HEK293 cells by replacing only 29 lipid-facing transmembrane L, I, V and F residues. CXCR4 was chosen because of its prominent role in 23 types of cancer metastasis and HIV infection^24–26^. Hence, a water-soluble variant could have potential therapeutic benefits, such as binding free CXCL12 to prevent cancer metastasis. Moreover, CXCR4’s ligand CXCL12 (SDF1α) and binding affinity are well characterized, and its crystal structure has been determined^27^, making it possible to make specific QTY replacements.

CXCR4^QTY29^ was indeed more water-soluble than the native CXCR4 when expressed in a cell-free system. The secondary structures of the purified native and QTY variants were comparable, as were the ligand-binding affinities. Most importantly, CXCR4^QTY29^ was able to signal through the G protein G_αq_ in HEK293 cells, albeit at ~ 4× higher CXCL12 ligand concentrations. Our results demonstrate that the QTY code can be used to design biologically functional hydrophilic GPCRs. Furthermore, the QTY code can likely be applied to other helical membrane proteins, thus opening the possibility for a wide range of biotechnological or medical uses.

## Results

### Computational modeling of CXCR4^QTY29^

Since we currently have not yet obtained the crystal structure of CXCR4^QTY29^, we tested whether CXCR4^QTY29^ is stable by simulating it in an explicit water environment at 24.85 °C, pH7.4, and 0.9% NaCl for 1 µs. After an initial 0.3 µs using the AMBER14 force field software, the overall structure was already formed and seemed to be stable. Additional 0.7 µs simulations did not further stabilize the structure (Supplementary Information). After the simulations, CXCR4^QTY29^ was superimposed with the crystal structure of the natural receptor (Fig. 1). The comparison showed that CXCR4^QTY29^ and CXCR4 have very similar structures. This indicates that the mutations do not adversely affect the protein structure when compared to the native receptor. The structural conservation observed further suggests that the QTY substitutions would not significantly affect the fold of the α-helices, and perhaps would not drastically affect the interaction of the ligand in the binding domain.

### Expression and water-solubility of CXCR4^QTY29^

**Figure 2:**
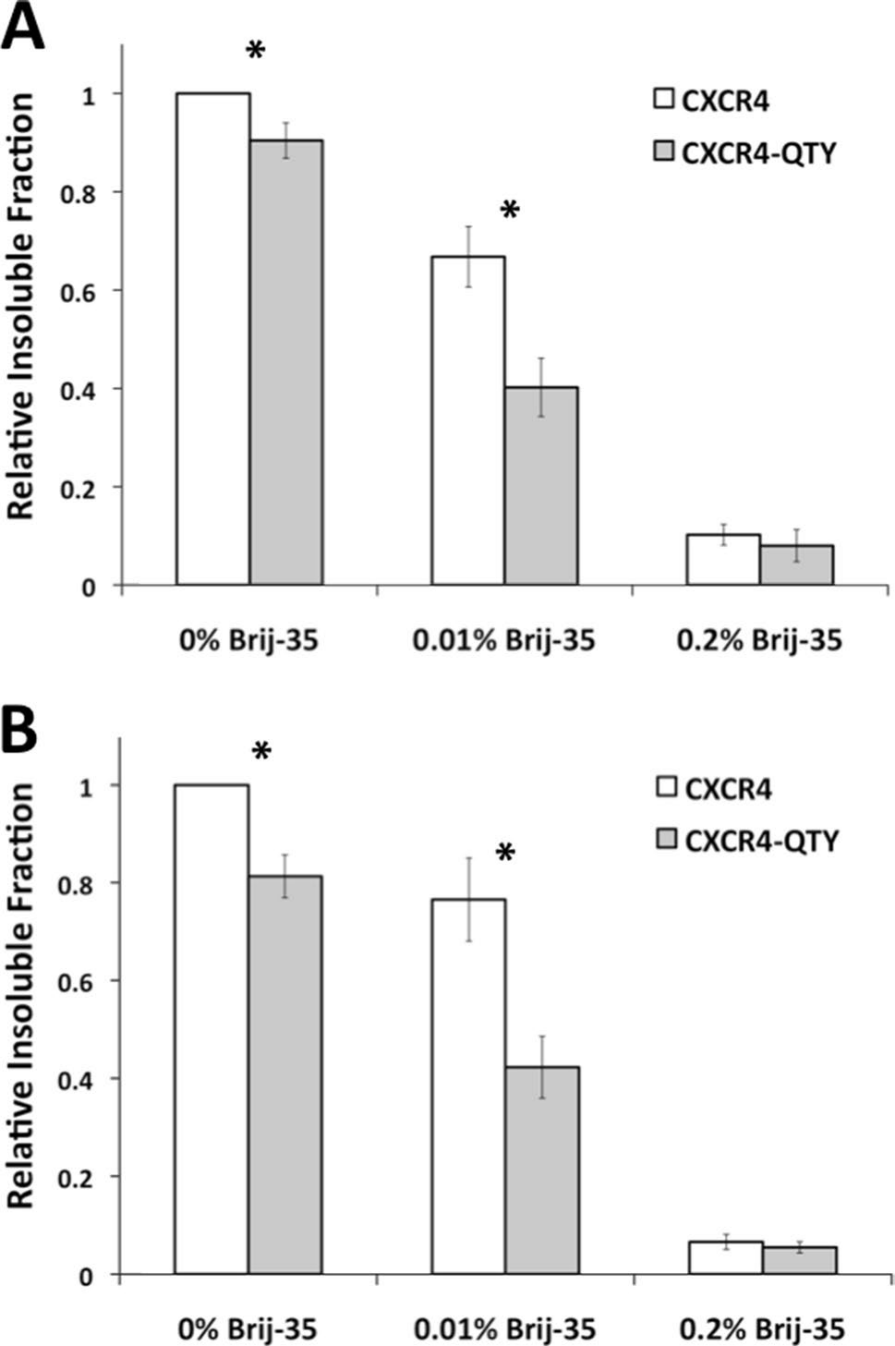
Relative insoluble fractions of CXCR4 and CXCR4^QTY29^ immediately after expression and 1 week later. (**A**) Insoluble fractions of CXCR4 and CXCR4^QTY29^ immediately after expression in 0%, 0.01%, and 0.2% Brij-35 (w/v). All data was normalized to CXCR4 in 0% Brij-35. Error bars show ± SEM. For 0% and 0.01% Brij-35, P < 0.01 (Student’s t-test, n = 12). (**B**) Insoluble fractions of CXCR4 and CXCR4^QTY29^ 1 week after expression in 0%, 0.01%, and 0.2% Brij-35 (w/v). All data was normalized to CXCR4 in 0% Brij-35. Error bars show ± SEM. For 0% and 0.01% Brij-35, P < 0.01 (Student’s t-test, n = 12).

*Escherichia coli*-based cell free extracts were used to express native CXCR4 and CXCR4^QTY29^. The detergent Brij-35 was used during expression because it has been previously demonstrated to be the optimal detergent for cell-free expressed GPCRs^28^. Both receptors were expressed in the following concentrations of Brij-35 (w/v): 0%, 0.01%, and 0.2%. As expected, native CXCR4 was most soluble at a Brij-35 concentration of 0.2%, and least soluble without detergent (Fig. 2). While CXCR4^QTY29^ showed a similar pattern of water-solubility, it was more soluble than the native CXCR4 at 0% and 0.01% Brij-35 (p < 0.01). This suggests that, although CXCR4^QTY29^ is not completely water-soluble, it appears to be more hydrophilic and more water-soluble than native CXCR4.

We then assayed the ability of CXCR4 and CXCR4^QTY29^ to remain water-soluble over time since it is known that most membrane proteins are not very stable long-term even in detergents. For these experiments, the expressed receptors were stored at 4 ºC for 1 week, after which they were centrifuged to remove any insoluble protein fractions. This insoluble fraction was compared to the fraction that remained soluble. As expected, the highest insoluble fraction was seen when no detergent was used, the lowest was seen with 0.2% Brij-35, and an intermediate insoluble fraction was observed at 0.01% Brij-35 (Fig. 2). Interestingly, Brij-35 at a concentration of 0.2% was able to keep nearly 100% of both receptors soluble for 1 week (Fig. 2). At Brij-35 concentrations of 0.01% and 0%, CXCR4^QTY29^ was significantly more water-soluble than native CXCR4 (p < 0.01). This observation is similar to the results immediately after in vitro translation. This suggests that, while CXCR4^QTY29^ is not fully water-soluble, it is more soluble than the native receptor. Furthermore, it is able to remain more water-soluble for 1 week.

### Purification of CXCR4^QTY29^

**Figure 3:**
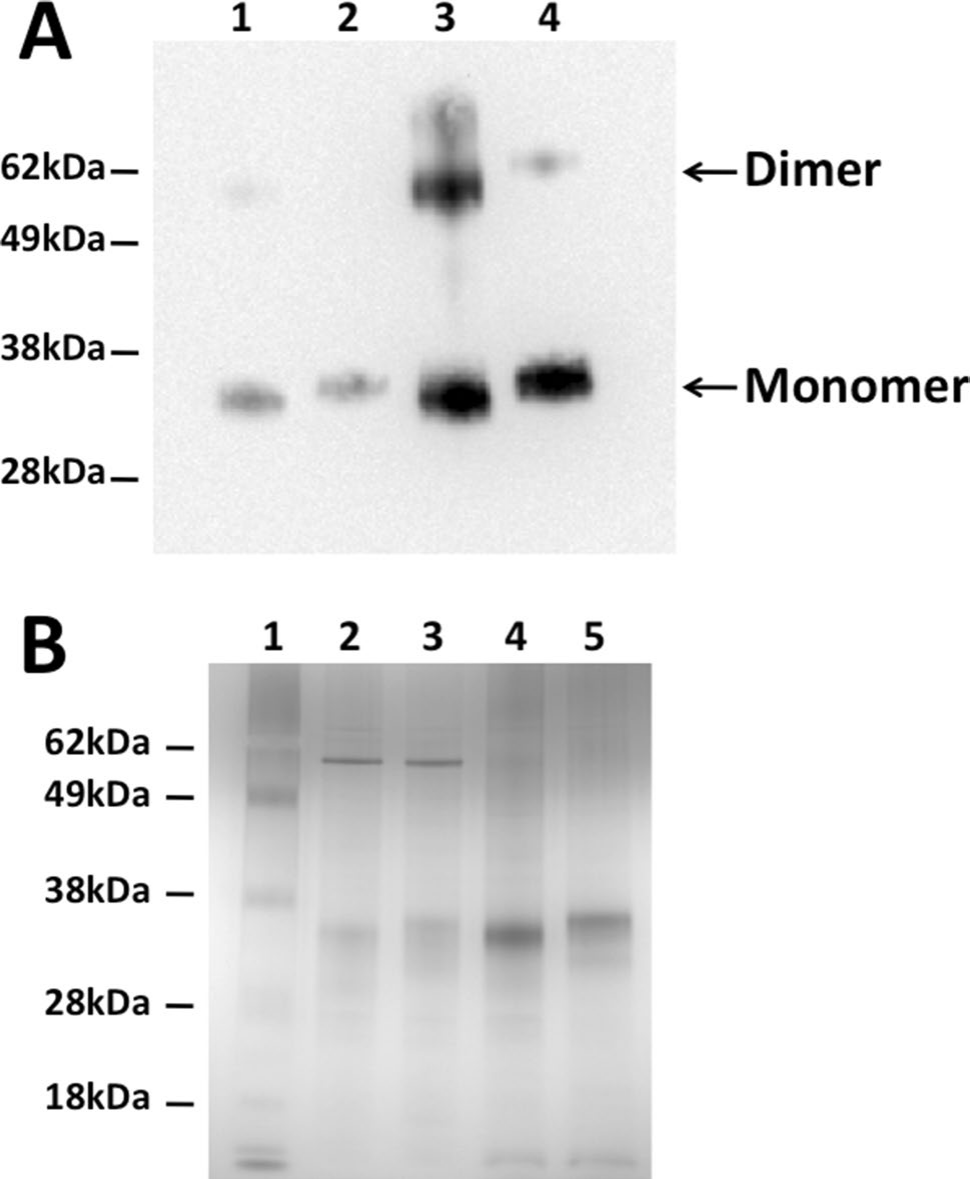
Western blot using a ρ1D4 monoclonal antibody, and silver stain of CXCR4 and CXCR4^QTY29^ in 0.01% and 0.2% Brij-35. (**A**) Western blot of CXCR4 and CXCR4^QTY29^. Lane 1: CXCR4 in 0.01% Brij-35; Lane 2: CXCR4^QTY29^ in 0.01% Brij-35; Lane 3: CXCR4 in 0.2% Brij-35; Lane 4: CXCR4^QTY29^ in 0.2% Brij-35. Although dimers are visible in both CXCR4 and CXCR4^QTY29^, a higher percentage of the expressed CXCR4 dimerizes. (**B**) Silver stain of purified CXCR4 and CXCR4^QTY29^. Lane 1: Ladder; Lane 2: CXCR4 in 0.01% Brij-35; Lane 3: CXCR4^QTY29^ in 0.01% Brij-35; Lane 4: CXCR4 in 0.2% Brij-35; Lane 5: CXCR4^QTY29^ in 0.2% Brij-35.

Immunoaffinity chromatography was used to purify CXCR4 and CXCR4^QTY29^ expressed in 0.01% and 0.2% Brij-35. An antibody against the C-terminal rho-tag was used to detect both receptors on Western blots (Fig. 3A). CXCR4 ran at ~ 34 kDa, while CXCR4^QTY29^ ran at ~ 36 kDa. The predicted molecular weights are 40.9 kDa and 41 kDa, respectively. Although the observed molecular weights are smaller than the predicted molecular weights, the blots likely show full-length receptor expression: membrane proteins typically run smaller than their predicted molecular weights^13,28^, the relative weights of CXCR4 and CXCR4^QTY29^ are as expected, and detection was against the C-terminal rho-tag. Silver stains were used to assay the purity of the receptors (Fig. 3B). After immunoaffinity chromatography alone, the receptors were typically ~ 90% pure, which was sufficient for downstream analyses. Interestingly, while Western blotting and silver staining indicated that both receptors could dimerize, this tendency was reduced with CXCR4^QTY29^. This is not surprising since the CXCR4^QTY29^ variant most likely disrupted or changed the dimerization interface^27^.

### Secondary structure analysis

**Figure 4:**
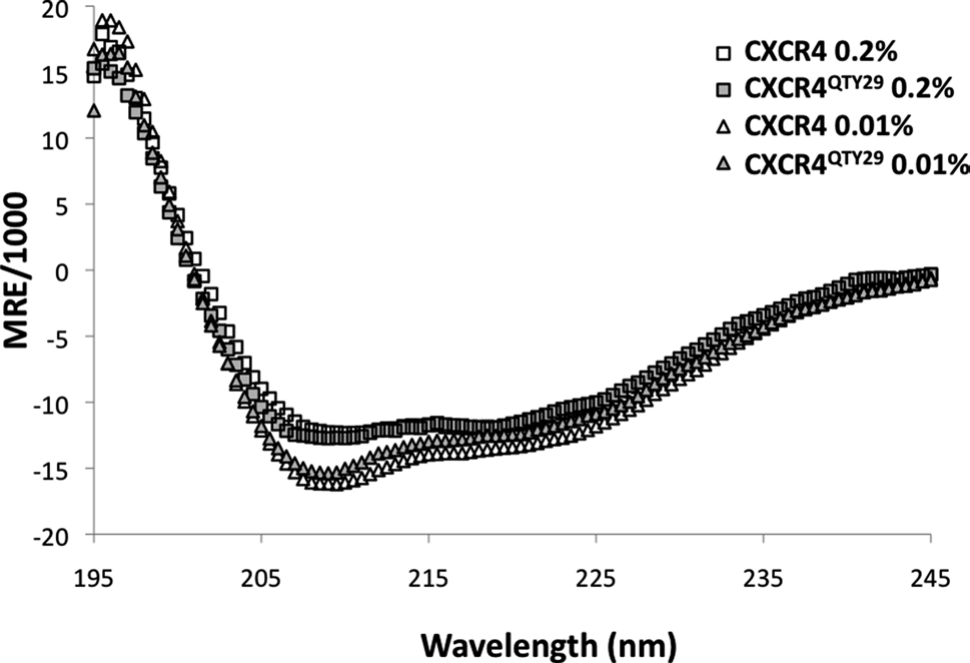
Circular dichroism spectra of CXCR4 and CXCR4^QTY29^ expressed in 0.01% or 0.2% Brij-35. All spectra have minima at ~ 208 nm and ~ 220 nm, which are characteristic for alpha-helical secondary structures. The spectra for receptors in 0.2% Brij-35 nearly overlap, suggesting nearly identical secondary structures. However, the spectra for the receptors in 0.01% Brij-35 are similar but distinct.

The secondary structures of CXCR4 and CXCR4^QTY29^ were analyzed using circular dichroism. The spectra of both receptors in the presence of 0.01%, and 0.2% FC14 were recorded. All samples exhibited characteristic alpha-helical spectra, with minima at ~ 208 nm and ~ 220 nm (Fig. 4). Because GPCRs have seven transmembrane alpha-helices, these results are expected thus suggesting proper folding. However, the spectra for CXCR4 and CXCR4^QTY29^ in 0.2% Brij-35 are different from the spectra in 0.01% Brij-35. Moreover, the spectra for receptors in 0.2% Brij-35 nearly overlap, while the spectra for receptors in 0.01% Brij-35 are similar but distinct. This suggests that there may be subtle differences in the secondary structures of CXCR4 and CXCR4^QTY29^ in 0.01% Brij-35, and that these structures are different from the secondary structures of both receptors in 0.2% Brij-35. However, it is also possible that any differences in the spectra are due to slight impurities. Taken together with the water-solubility data, the circular dichroism data suggests that the presence of detergent keeps the receptors properly folded, and that the structure of CXCR4^QTY29^ at 0.01% Brij-35 is slightly more similar to the structure in 0.2% Brij-35 than that of the native receptor.

### Ligand-binding analysis

**Figure 5:**
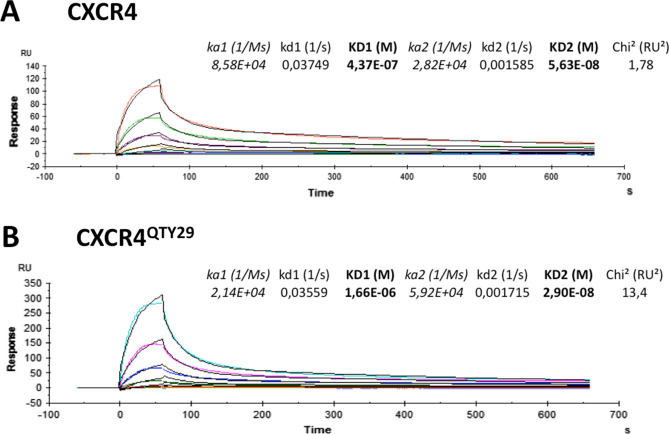
Surface Plasmon Resonance data of the interaction between SDF1α and (**A**) CXCR4 or (**B**), CXCR4^QTY29^. Colored lines represent the experimental data, while black lines represent fitted curves using the heterogeneous ligand-binding model in the BiaEvaluation program. Parameters from the fitting are shown in-line. The closeness of the fit, described by the statistical value χ^2^ (Chi^2^), was 1.78 for CXCR, and 13.4 for CXCR4^QTY29^, indicating that the model used is slightly more true to reality for CXCR4. The high-activity fractions of CXCR4^QTY29 ^and wild-type CXCR4 have similar affinities and binding kinetics. The low-activity fraction of CXCR4^QTY29^ has a similar binding kinetic profile as CXCR4, but has a higher association rate constant yielding a lower affinity to the ligand. The active fractions of both receptors have comparable affinities to SDF1α. The K_D_ values measured in this work are ~ 29 nM (CXCR4) and ~ 56 nM (CXCR4^QTY29^), which are comparable to reported CXCR4 affinities ranging from 5 to 200 nM^29,30^.

We asked if CXCR4^QTY29^ interacts with the ligand CXCL12 (SDF1α) in the same fashion as the native CXCR4. Surface Plasmon Resonance (SPR) was used to carry out the ligand binding measurements. Both receptors were captured in different flow cells using the Rho-tag or 1D4 antibody immobilized on the surface, as previously described^29^. The same ligand preparations were first flowed over CXCR4, and were then flowed over CXCR4^QTY29^ for comparison. To obtain a reasonable fit to the SPR data, both CXCR4 and CXCR4^QTY29^ were analyzed with the “heterogenous ligand” method. The results suggest that the cell free protein expression system yields one protein fraction with lower activity, and one fraction with higher activity. Nevertheless, the more active fractions of both CXCR4 and CXCR4^QTY29^ have affinities that are similar to those previously reported for the natural CXCR4 isolated from cells. The K_D_ values measured in this work are ~ 29 nM and ~ 56 nM, respectively, compared to reported CXCR4 affinities ranging from 5 to 200 nM^29,30^, depending on the preparation methods used (Fig. 5). Most importantly, the cell-free produced CXCR4^QTY29^ binds CXCL12 with a similar interaction profile as the cell-free produced wild type CXCR4, suggesting that the ligand CXCL12 interacts with both receptors in a similar manner.

### CXCR4 and CXCR4^QTY29^ localization in HEK293 cells

**Figure 6:**
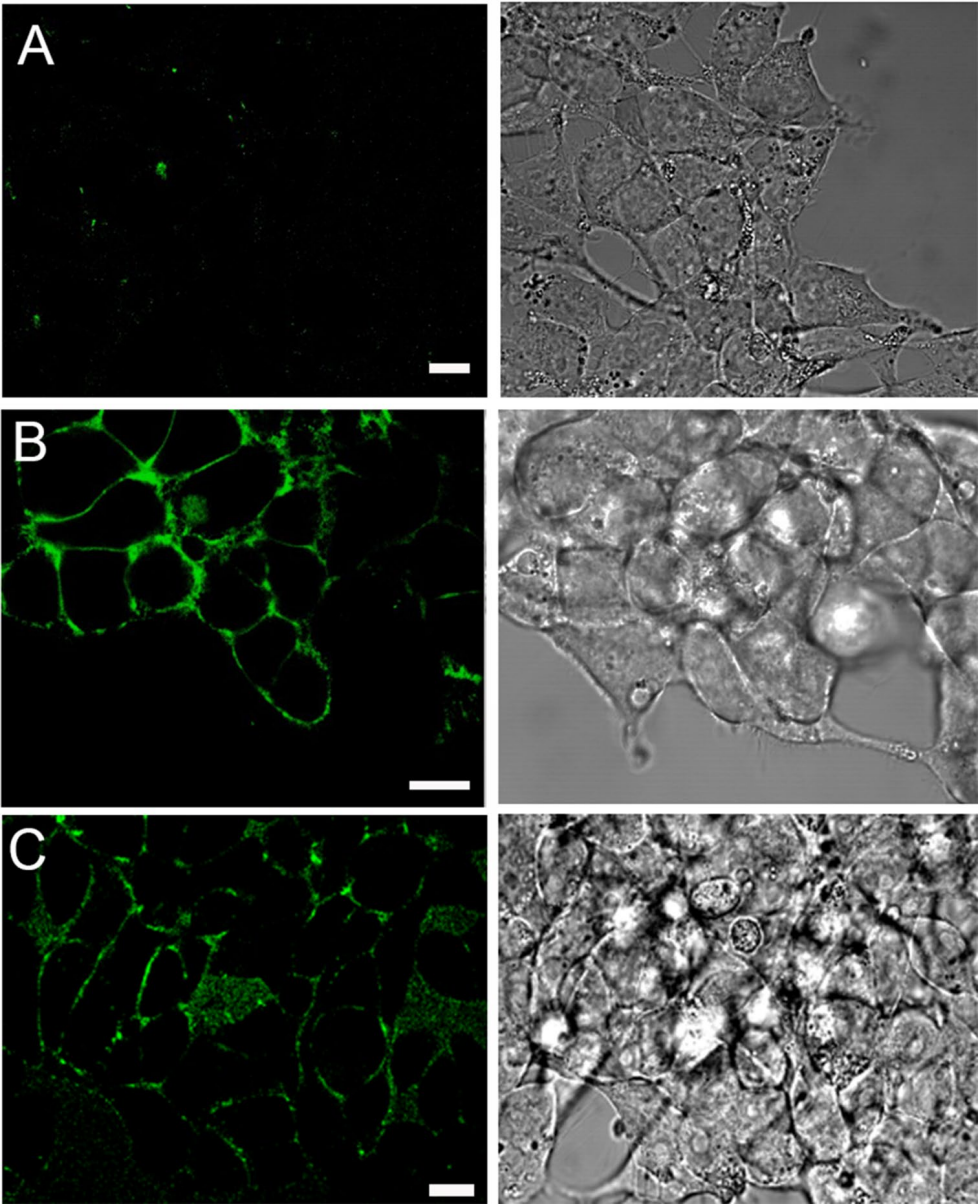
Laser-scanning confocal micrographs showing fluorescently-labeled cell surface expression of wild type CXCR4 and CXCR4^QTY29^. Left hand: fluorescence images (Ex. 488 nm/Em. 505 nm, LP filter). Right hand: transmission images. (**A**) Negative control, showing stable HEK293 cells not induced for CXCR4 expression. (**B**) HEK293 cells expressing CXCR4 wildtype. (**C**) HEK293 cells expressing CXCR4^QTY29^. Equal amounts of CXCR4 and CXCR4^QTY29^ were transfected into HEK293 cells in parallel and under identical conditions. These images indicate that CXCR4 and CXCR4^QTY29^ traffic to the cell membrane, but that the amount of membrane-bound CXCR4^QTY29^ is less than that of CXCR4. The size bar is 10 mm.

**Figure 7:**
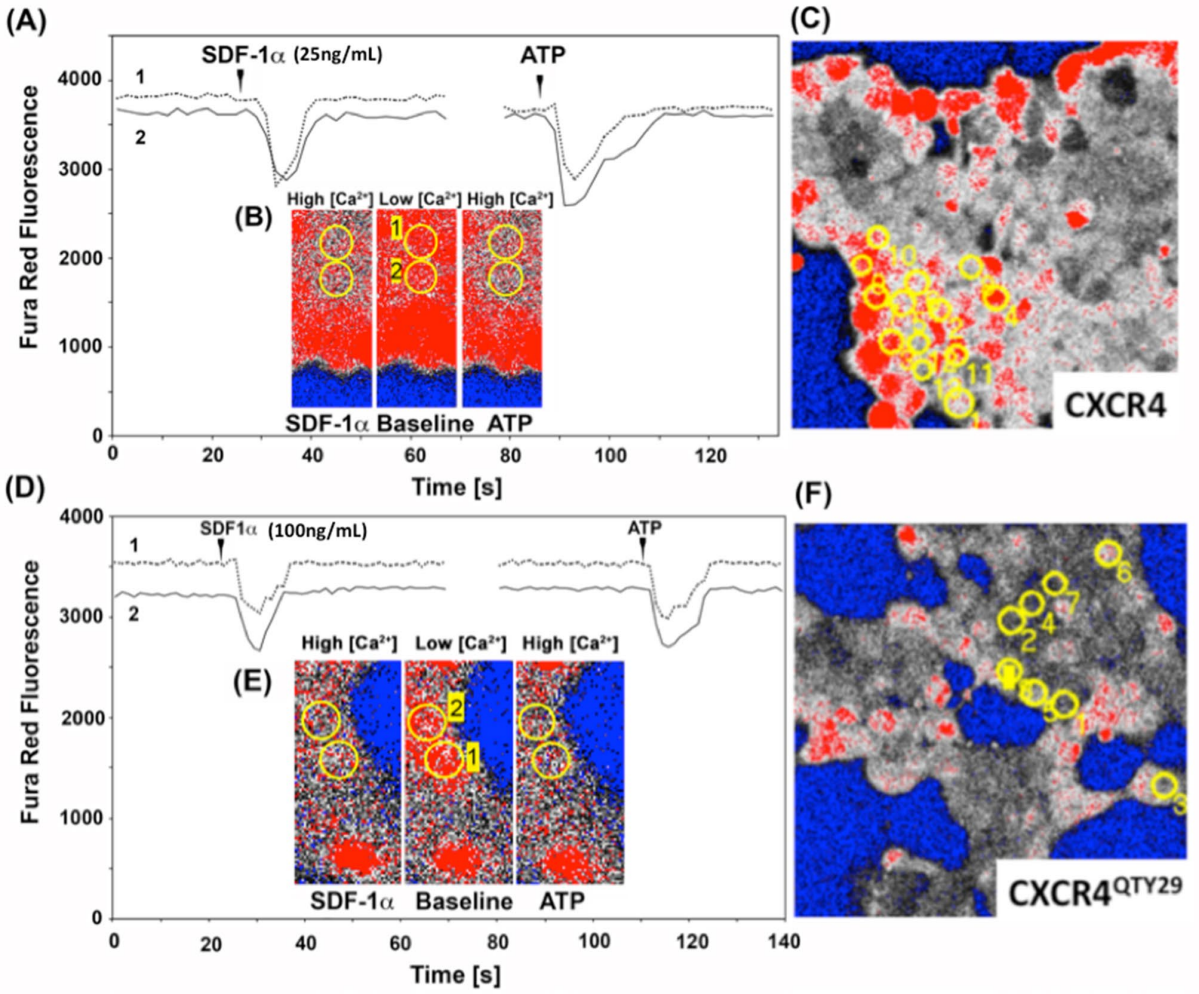
Intracellular calcium signaling in CXCR4- or CXCR4^QTY29^-transfected HEK293 cells in response to the ligand SDF-1α. CXCR4 or CXCR4^QTY29^ were co-transfected with the G-protein G_aq_ into HEK293 cells, and the cytosolic Ca^++^ concentration in response to SDF1α was monitored with FuraRed. (**A**)–(**C**) In CXCR4-transfected cells, the intracellular calcium response to application of 25 ng/ml (~ 3.2 nM) SDF1α and subsequent application of ATP (100 mM) as a viability control were recorded. (**D**)–(**F**) In CXCR4^QTY29^-transfected cells, the intracellular calcium response to application of 100 ng/ml (~ 13 nM) SDF1α and the subsequent application of ATP (100 mM) as a viability control were recorded. The fluorescence traces in (**A**) and (**D**) show the response of a single cell to both reagents. (**B**) and (**E**) show zoomed fields of transfected cells. (**C**) and (**F**) Show the frequency of individual Ca^2+^ responses in populations of CXCR4- and CXCR4^QTY29^-transfected HEK293 cells in response to SDF1α. Circles highlight active cells. In both cases, PBS or mock-transfected cells were used as negative controls, and the fluorescence in response to SDF1α was indistinguishable from baseline, while ATP could still elicit a normal response.

Since CXCR4^QTY29^ still has hydrophobic residues in its 7 transmembrane α-helices, we assayed whether it could still insert itself into the cell membrane. In order to visualize where the expressed receptors were localized, HEK293 cells from the same parent population were transfected in parallel with the genes that express either CXCR4 or CXCR4^QTY29^. Immunohistochemical staining was then performed to locate the expressed receptors. Both receptors trafficked to the cell membrane (Fig. 6). However, significantly more of the native CXCR4 was visualized in the membrane than CXCR4^QTY29^. Because CXCR4^QTY29^ is designed to be more water soluble, it is possible that less CXCR4^QTY29^ is able to remain tightly embedded in the lipid bilayer. It is also possible that it is less capable of effectively inserting into the cell membrane. This difference in cell-surface receptor concentration is likely to be responsible for the reduced cell-signaling response observed in CXCR4^QTY29^-transfected HEK293 cells (Fig. 7).

### Cell-signaling of CXCR4^QTY29^ in HEK293 cells

The biologically functional activity of CXCR4^QTY29^ was assayed by co-transfecting it with the G-protein G_aq_ into HEK293 cells. For comparison, the native CXCR4 was co-transfected with G_aq_ into HEK293 cells in parallel and under identical experimental conditions. Both CXCR4 and CXCR4^QTY29^-transfected cells showed a transient increase in intracellular calcium upon application of the ligand CXCL12 (SDF1α) (Fig. 7). However, native CXCR4 seemed more readily able to initiate signal transduction than CXCR4^QTY29^: a strong response was seen from native CXCR4 at a CXCL12 (SDF1α) concentration of 25 ng/ml (~ 3.2 nM), while 100 ng/ml (~ 13 nM) CXCL12 typically needed to be applied to CXCR4^QTY29^ in order to observe a similar response. This concentration reflects a fourfold increase. Moreover, it appears that more cells expressing native CXCR4 signaled upon application of CXCL12 (SDF1α) than cells expressing the QTY variant. The decreased signaling response seen from CXCR4^QTY29^ likely reflects a lower concentration of biologically available receptors as demonstrated via membrane localization experiments (Fig. 6). However, it is also possible that the reduced signaling is also due to disruption of the dimer interface, or nuanced changes in the binding pocket.

ATP was used as a positive control, while PBS and mock-transfected cells were used as negative controls (Fig. 7). After application of CXCL12 (SDF1α), the calcium levels were allowed to return to baseline levels, after which the cells were stimulated with ATP. In each case, a signaling response was observed. PBS was added to the cells either before or after CXCL12 (SDF1α) was applied. No change in calcium concentration was observed in both cases. Similarly, mock-transfected cells did not respond to either PBS or CXCL12 (SDF1α), but did respond to ATP.

Taken together, this data clearly shows that CXCR4^QTY29^ is capable of signaling in response to the native ligand CXCL12 (SDF1α), albeit in a fourfold reduced signaling capability. This further suggests that the CXCR4^QTY29^ variant did not completely disturb the ligand-binding domain, and that CXCR4^QTY29^ is likely properly folded, integrated into the membrane though perhaps less efficiently, and is functional.

## Discussion

In spite of the recent expansion in GPCR structures^31^, the number of known structures is still miniscule compared to the size and importance of this receptor family. Indeed, GPCRs comprise > 2% of the human genome and include 825 different types of receptors^6,32^. Moreover, they are the targets of ~ 50% of pharmaceutical drugs, which are used to treat diseases ranging from hypertension, congestive heart failure, coronary heart disease, asthma, pre-term labor, schizophrenia, Parkinson’s disease, HIV, cancer, and diabetes mellitus-induced renal damage^33^. Yet, the structures of only 88 receptors have been determined. This relatively small number of determined structures is solely due to the difficulty of expressing sufficient amounts of soluble, stable, and functional receptors for structure and function assays.

It is necessary to determine the molecular structures of GPCRs in order to understand how they function at the molecular level, and to design specific drug targets, clinical therapies, and innovative protein-based technologies. Almost all of the crystallized GPCRs needed to be modified in order to enable sufficient expression and to facilitate crystallization. In spite of these modifications, detergents were still required, which have been shown to hamper crystallization^34^. Moreover, disordered regions still existed where the structure could not be determined, and the most successful strategy (insertion of T4L) can interfere with the ability of the modified GPCRs to signal.

The QTY code we present here is a simple alternative method of obtaining GPCRs suitable for structure and function studies that offers advantages over previous approaches. We have shown that the QTY code can be used to design a more hydrophilic form of the receptor CXCR4 that is biologically active, and properly folded and functional after purification. Because only the helix portions facing the membrane are changed, the designed receptor is capable of signaling through G-proteins upon ligand-binding. And unlike most previous GPCR modifications and computer-modeling to make water-soluble variants, this method reduces the need for detergents. This opens the door for diverse applications including sensing devices, the development of decoy therapies, and beyond.

We have previously shown that the QTY code can be used to generate completely water-soluble receptors. However, this required a significant number of modifications: those receptors had > 20% mutations, with ~ 50% residue replacements in the transmembrane regions^23^. Moreover, cell-signaling and trafficking assays were not performed, so it was unknown whether QTY-modified receptors could still insert into the membrane or remain biologically active. Here, we were able to design a more hydrophilic receptor that is capable of inserting into the cell membrane and signaling. This variant receptor had only 29 residues replaced (~ 8.2%), and these lipid-facing residues were targeted and rationally designed. Of significance, our data indicates that the mutant CXCR4^QTY29^ retains some cell surface expression in HEK293 cells. This suggests that CXCR4^QTY29^ folding, intracellular trafficking, and membrane insertion is preserved to some extent. Without proper protein folding, the receptor would accumulate in the endoplasmic reticulum, and receptor translocation to the surface plasma membrane would be arrested^35^. Furthermore, cell surface expression is a critical prerequisite for cell signaling. Our confocal images indicate that the ligand-binding site of CXCR4^QTY29^ is accessible on the surface of HEK293 cells as it binds an externally added, receptor-specific ligand. A qualitative comparison of HEK293 cells expressing wild-type CXCR4 or CXCR4^QTY29^ indicates visibly weaker expression of the QTY receptor. This implies that, while binding is preserved, the QTY modification has some influence over cell surface expression. This is in agreement with the SPR experiments reported in this study.

The QTY code described here will likely facilitate more structure and function studies since more water-soluble receptors can be designed without the need for complex computational analyses or extensive library screens. This will not only aid in structural studies, but may also make expression easier. Moreover, it may aid in the design of other helical transmembrane proteins.

The principal significance of the QTY code is that it is a simple and potentially general protein design approach that can be applied to other GPCRs without the need for extensive computational calculations. Moreover, this QTY code can likely be applied to other alpha-helical transmembrane proteins as well. And, because the resulting receptor is more water-soluble, it can not only be used for structure and function studies, but also has the potential to be used in novel technological developments requiring water-soluble receptors or novel protein designs.

## Methods

### CXCR4^QTY29^ gene and protein design

As a GPCR, CXCR4 is characterized by 7 transmembrane alpha helices arranged in a barrel-like conformation. The inside of this barrel is more hydrophilic, while the outside contacting the lipid bilayer is hydrophobic. Loops connecting the helices are located either inside or outside the cell membrane. Only residues in the helical transmembrane segments facing the lipid bilayer were replaced with hydrophilic QTY residues (Fig. 1) Residues in the external loops that interact with the ligand were not changed. Seven amino acids have higher helical forming tendencies: leucine (L), glutamine (Q), threonine (T), phenylalanine (F), and tyrosine (Y). Q, T, and Y can form hydrogen bonds and are more hydrophilic than L, I, V, and F, but they bear no positive or negative charges. Moreover, the following pairs have similar shapes and sizes: L and Q, V or I and T, and F and Y. In our proposed scheme, Q replaced L, T replaced V or I, and Y replaced F (hence QTY).

To design CXCR4^QTY29^, the crystal structure of CXCR4 (PDB 3ODU) was visualized using VMD software^36^ (http://www.ks.uiuc.edu/Research/vmd/), and the QTY method above was applied to residues facing the lipid bilayer. A total of 29 residues (8.2% of the total amino acid sequence) were changed: F36Y, I39T, F49Y, L50Q, I53T, V82T, L86Q, I89T, F93Y, V96T, V99T, I162T, L165Q, I169T, F172Y, I173T, V242T, I243T, L246Q, L253Q, I257T, F264Y, I286T, L290Q, F293Y, L297T, I300T, L301Q and F304Y.

The protein sequence for the natural CXCR4 (P61073) was obtained from Uniprot (http://www.uniprot.org/uniprot/P61073), and the protein sequence for CXCR4^QTY29^ was designed as described above and ordered from Geneart (Thermo Fisher Scientific, Waltham, MA). Both sequences were optimized for expression in *E. coli*. A C-terminal rho tag was added for purification, and the constructs were cloned into the pIVex2.3 vector for cell-free expression, and the pcDNA4T/O vector for cell-signaling assays. The genes were amplified using Qiagen (Germantown, MD) plasmid mini and maxiprep kits. The full final sequence of CXCR4^QTY29^ is shown in Fig. 1.

### Computer simulations of CXCR4 and CXCR4^QTY29^

The structure for CXCR4 was obtained from the Protein Data Bank (code 3ODU, chain A). An initial structure for CXCR4^QTY29^ was obtained from the predicted sequence and the GOMoDo modeling server^37^. The simulations were done in an explicit water environment at 24.85 °C, pH7.4, and 0.9% NaCl for 1 µs using the full-atom AMBER14 N self-parameterizing force field within the simulation software YASARA^38^. The MD was simulated at a 5 fs timestep using a simulation cell 20 Å larger than each candidate, but with re-centering of the candidate at each timestep, and with generation and removal of water at the boundary to negate the possibility of non-physical boundary effects^39^. The CXCR4^QTY29 ^model was then aligned with its detergent-encapsulated counterpart CXCR4 using MUSTANG48 and superimposed^40^. The computer used for the simulations was built with an Intel Core i7-6950X10-Core 3.0 GHz Processor, GIGABYTE GeForce GTX 1080 Video Card, and 16 GB of DDR4 2800 memory.

### Cell-free protein expression

Cell-free expression has been previously described^28^. Briefly, native CXCR4 or CXCR4^QTY29^ DNA was added to a commercial *E. coli* cell-free reaction mix (ThermoFisher, Qiagen, or RiNA) according to the manufacturer’s instructions. The detergent Brij-35 was added at concentrations ranging from 0 to 0.2% (w/v). The completed reactions were centrifuged at 10,000 rpm for 10 min. The supernatant with the solubilized receptor was removed, and the pellet was resuspended in an equivalent volume of PBS. The relative quantities of solubilized and precipitated protein were assayed with a dot blot and analyzed using ImageJ^41^.

### Immunoaffinity purification

CNBr-activated Sepharose 4B beads (GE Healthcare) coupled to the rho1D4 monoclonal antibody (Cell Essentials, Boston, MA) were used to purify CXCR4 and CXCR4^QTY29^ as previously described^28^. Briefly, solubilized protein was captured on the beads overnight at 4 °C. The beads were washed with wash buffer (PBS + 0.01% or 0.2% FC14) until all impurities had been removed. The receptor was eluted from the beads with elution buffer (PBS + 0.01% or 0.2%FC14 + 800 µM elution peptide). The eluted protein was concentrated using 50 kDa MWCO (0.2% detergent samples), 30 kDa MWCO (0.2% or 0.01% detergent samples), or 10 kDa MWCO (0.01% detergent samples) filter columns. To remove excess elution peptide, the samples were washed with ~ 67 × volumes of wash buffer in the centrifugation filter columns at 4 ºC, 1000–1200 g, for intervals of 1–2 min. The centrifugation filter columns were from Millipore (Burlington, MA), and the elution peptide Ac-TETSQVAPA-CONH_2_ was synthesized by CPC Scientific, Inc (Sunnyvale, CA).

### CXCR4 and CXCR4^QTY29^ detection and purity analysis

Dot blots were used to analyze the relative solubility of different samples, while western blots and silver stains were used to detect the receptors and analyze their purity as previously described^42,43^. Samples were loaded into Novex 10% Bis–Tris Gels (Life Technologies, Waltham, MA) according to the manufacturer’s instructions, with the exception that the samples were incubated at room temperature for 10 min as boiling can cause receptor aggregation. After electrophoresis, the samples were transferred to a nitrocellulose membrane, blocked with milk (5% w/v non-fat dried milk in TBST) for 1 h, and incubated with the rho1D4 monoclonal antibody (1:3000 in TBST, 1 h at room temperature or overnight at 4 ºC). A goat anti-mouse HRP-conjugated secondary antibody (Pierce, Rockford, IL) and the ECL-Plus Kit (GE Healthcare, Pittsburgh, PA) were used to visualize the receptors. The SilverXpress kit (Life Technologies) was used according to the manufacturer’s instructions to do total protein stains. All images were captured on a Biorad GelDoc system. ImageJ was used to compare sample intensities and to analyze sample purity.

### Secondary structure analysis using circular dichroism

Circular dichroism (CD) spectra were recorded at 15 ºC from 195 to 245 nm with a step size of 1 nm, and an averaging time of 4 s (Aviv Biomedical, model 410, or Jasco J-1500). The spectra were blanked to wash buffer, and represent an average of 3–5 repeat runs. A quartz sample cell with a path length of 1 mm was used. 300 µL of receptor sample were used for each experiment.

### Ligand-binding analysis

SPR analyses were performed with Biacore T200 and Biacore 2000 instruments (GE Healthcare). Biacore CM4 series S chips were used, and the ID4 antibody was immobilized on the chips using standard EDC/NHS coupling chemistry. Immobilization was performed with 50 mg/ml 1D4 antibody in 10 mM Acetate buffer, pH 5.0 and contact times between 5 and 7 min to achieve immobilization levels of 8000–12,000 RU. The chemokine receptors CXCR4 and CXCR4^QTY29^ were captured to 2500–5000 RU from crude supernatants from cell free reactions, and the surface was washed with PBS supplemented with 0.2% FC-14 until the surface bleeding was less than 1 RU/min (40–180 min). All experiments were performed at 25 °C in 50 mM Hepes (pH 7.5), 5 mM MgCl_2_, 1 mM CaCl_2_, and 1 mg/ml BSA. The natural ligand SDf1α (7.8 kDa) was flown over the surfaces at concentrations of 0, 3.125, 6.25, 12.5, 25, 50, 100, 200 and 400 nM. The injections were made in triplicate with a blank injection before each injection due to slow dissociation. Analyses were made in BiaEvaluation software (GE Healthcare).

### Cell-signalling assays

HEK293 cells derived from a single culture were seeded onto 0.18 mm thick glass slides at a density of 0.5 × 10^6^ cells per mL and were grown overnight at 37 °C in the incubator to allow adhesion of the cells to the surface of the slides. In parallel and under identical conditions, the cells were transiently transfected with G_αq_ and either CXCR4 or CXCR4^QTY29^ DNA using Lipofectamine2000 (Invitrogen) according to the manufacturer’s instructions. Six hours after transfection, the cells were induced with doxycyclin. Eighteen to 20 h after induction, the cells were washed with PBS and loaded with 10 µM Fura-Red-AM (ThermoFisher Scientific, Waltham, MA) for 30 min in serum-free DMEM/F12 medium. This medium was then replaced with fresh DMEM supplemented with 10% serum, and the cells were incubated an additional 30 min before imaging. Calcium signaling in response to 25 ng (for CXCR4) or 100 ng (for CXCR4^QTY29^) SDF1a was visualized using confocal fluorescence microscopy (Zeiss LSM 510) with a water immersion objective (Zeiss Achroplan 63 × NA 1.2). The cells were excited at 488 nm (Ar + laser), and emission was monitored at 650 nm. Images were collected every 2 s for a total of 100 s. ATP was used as a positive control, and PBS and mock-transfected cells were used as negative controls. The cells were incubated at 37 ºC and 5% CO_2_ in DMEM supplemented with 10% FCS (Life Technologies) unless noted otherwise.

### Cell-staining assays

HEK293 cells expressing CXCR4 or CXCR4^QTY29^ were grown on 0.18 mm thick cover glasses using a seeding density of 3 × 10^5^ cells/ml in DMEM/F12 medium containing 10% FCS (GIBCO). Visualization of cell surface-expressed receptors was achieved by labeling the cells with 1 mM receptor-specific peptide 6 (Alexa 488) in PBS buffer for 10 min. Cells were washed with PBS buffer to remove unbound ligand. Images were recorded using a 488 nm Ar/Kr laser line and a LP505 mm filter on a Zeiss LSM510 laser-scanning confocal microscope with a 63× water objective (1.2 numerical aperture).

## Supplementary information

Supplementary Information 1.

Supplementary Information 2.

Supplementary Information 3.

Supplementary Figures.
