# Supplementary material for: The G protein coupled receptor CXCR4 designed by the QTY code becomes more hydrophilic and retains cell signaling activity
Source: Sci Rep. 2020 Dec 7;10:21371. doi: 10.1038/s41598-020-77659-x (PMC7721705; doi:10.1038/s41598-020-77659-x)
Supplement: Supplementary file 4 — Supplementary Figures. [file 41598_2020_77659_MOESM4_ESM.docx]

Supplementary Information for

**The G protein coupled receptor CXCR4 designed by the QTY code becomes more hydrophilic and retains cell signaling activity**

Lotta Tegler^1, 2, ¶^, Karolina Corin*^1, 3, ¶, 6^, Horst Pick^4^, Jennifer Brookes^1,7,8^, Michael Skuhersky^5^, Horst Vogel^4^, Shuguang Zhang*^1^


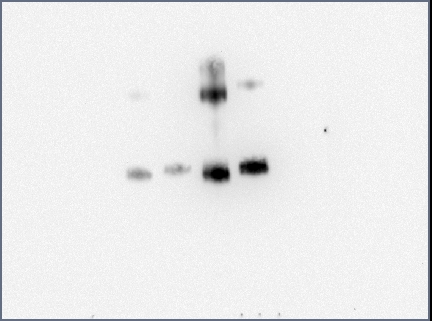


Supplementary Figure 1: Uncropped version of Figure 3A.


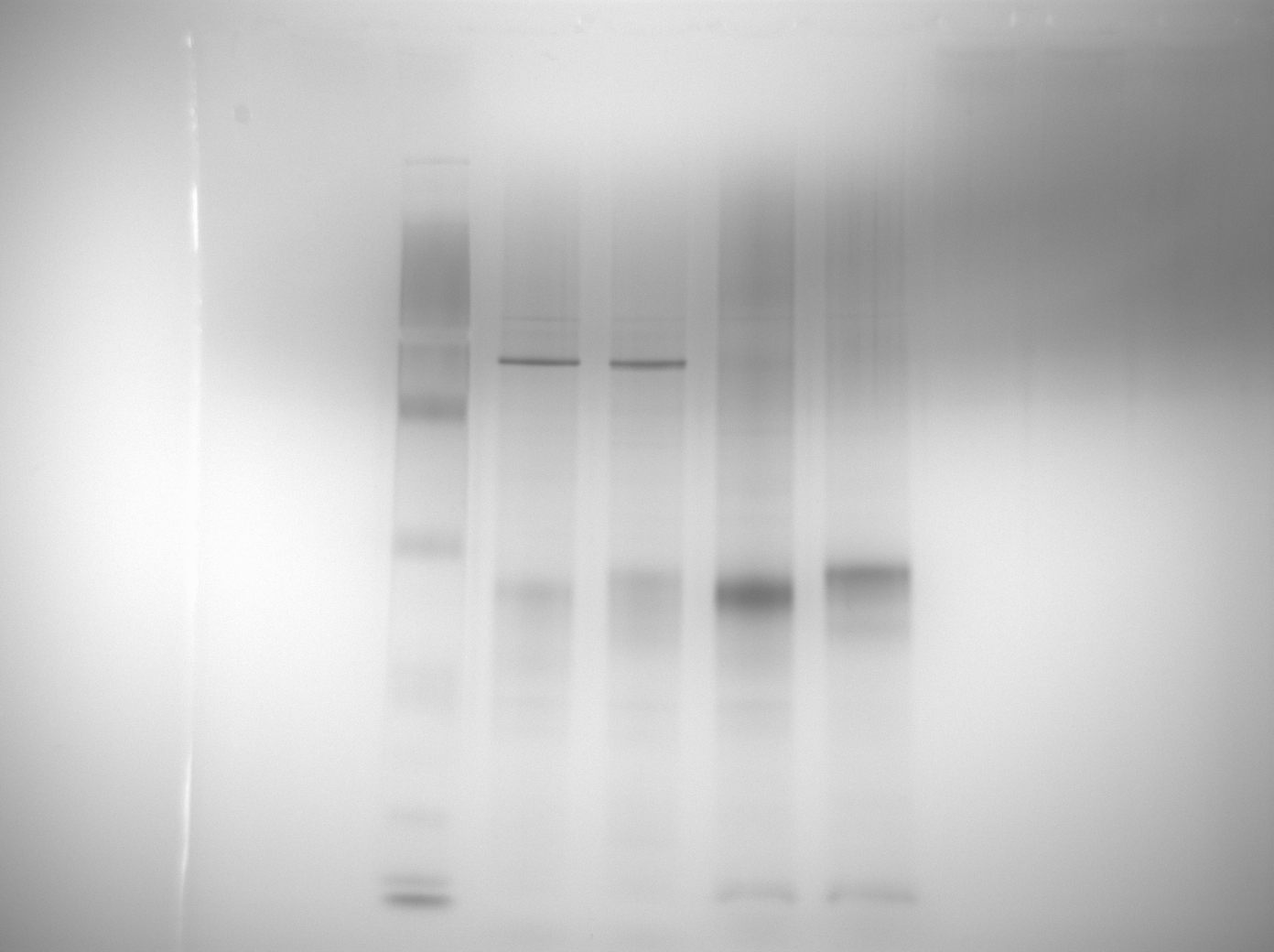


Supplementary Figure 2: Uncropped version of Figure 3B.
